# Processing and Maturation of Cathepsin C Zymogen: A Biochemical and Molecular Modeling Analysis

**DOI:** 10.3390/ijms20194747

**Published:** 2019-09-25

**Authors:** Anne-Sophie Lamort, Yveline Hamon, Cezary Czaplewski, Artur Gieldon, Seda Seren, Laurent Coquet, Fabien Lecaille, Adam Lesner, Gilles Lalmanach, Francis Gauthier, Dieter Jenne, Brice Korkmaz

**Affiliations:** 1 INSERM UMR-1100, CEPR (Centre d’Etude des Pathologies Respiratoires), 37032 Tours, France; anne-sophie.lamort@helmholtz-muenchen.de (A.-S.L.); yv.hamon@gmail.com (Y.H.); seda.seren@etu.univ-tours.fr (S.S.); fabien.lecaille@univ-tours.fr (F.L.); gilles.lalmanach@univ-tours.fr (G.L.); francis.gauthier@univ-tours.fr (F.G.); 2 Université de Tours, 37032 Tours, France; 3 Comprehensive Pneumology Center and Institute for Lung Biology and Disease; University Hospital, Ludwig-Maximilians University of Munich and Helmholtz Center Munich; Member of the German Center for Lung Research, 81377 Munich, Germany; dieter.jenne@helmholtz-muenchen.de; 4 Faculty of Chemistry, University of Gdansk, 80-308 Gdansk, Poland; cezary.czaplewski@ug.edu.pl (C.C.); artur.gieldon@ug.edu.pl (A.G.); adam.lesner@ug.edu.pl (A.L.); 5 CNRS UMR-6270, Normandie Université, Plate-forme PISSARO, 76821 Mont Saint Aignan, France; laurent.coquet@univ-rouen.fr; 6 Max Planck Institute of Neurobiology, 82152 Planegg-Martinsried, Germany

**Keywords:** Cathepsin C, cysteine cathepsin, zymogen, zymogen processing

## Abstract

Cysteine cathepsin C (CatC) is a ubiquitously expressed, lysosomal aminopeptidase involved in the activation of zymogens of immune-cell-associated serine proteinases (elastase, cathepsin G, proteinase 3, neutrophil serine proteinase 4, lymphocyte granzymes, and mast cell chymases). CatC is first synthetized as an inactive zymogen containing an intramolecular chain propeptide, the dimeric form of which is processed into the mature tetrameric form by proteolytic cleavages. A molecular modeling analysis of proCatC indicated that its propeptide displayed a similar fold to those of other lysosomal cysteine cathepsins, and could be involved in dimer formation. Our in vitro experiments revealed that human proCatC was processed and activated by CatF, CatK, and CatV in two consecutive steps of maturation, as reported for CatL and CatS previously. The unique positioning of the propeptide domains in the proCatC dimer complex allows this order of cleavages to be understood. The missense mutation Leu172Pro within the propeptide region associated with the Papillon–Lefèvre and Haim–Munk syndrome altered the proform stability as well as the maturation of the recombinant Leu172Pro proform.

## 1. Introduction

**Scheme 1:**
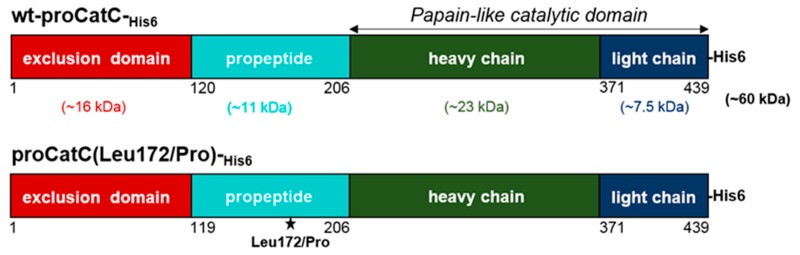
Schematic structure of human wt-proCatC and proCatC(Leu172Pro) produced in HEK293 EBNA cells. The numbering does not take into account the signal peptide and refers only to the proCatC sequence. Molecular masses for exclusion domain and heavy chain, observed by western blot detection, represent apparent masses with glycosylation.

Eleven cysteine cathepsins (B, C, F, H, L, S, K (a.k.a. O_2_), O, V, X (a.k.a. Z/P) and W) that are related to papain (clan CA, family C1, subfamily C1A; *see MEROPS: the peptidase database (Release 12.1): http://merops.sanger.ac.uk*) have been identified and characterized to date in humans [1,2,3]. They all are lysosomal proteinases, which not only act as house-keeping proteinases involved in bulk protein degradation, but also process other endogenous proteins in a relatively specific manner [1,2]. Cathepsin C (CatC), also known as dipeptidyl peptidase I (EC 3.4.14.1), is a unique member of the cathepsin family in view of its elaborate biosynthesis and homo-tetrameric structure [4,5]. CatC is initially synthesized as a ~60 kDa single chain glycosylated monomer (_1_Asp–Leu_439_), that associates to form the proCatC homodimer [4]. Proteolytically active, mature CatC is generated by cleavages at several sites in each monomer, resulting in an almost complete excision of the internal propeptide (_120_Thr–His_206_) and the appearance of the three chains of mature CatC, i.e., the exclusion domain (_1_Asp–Gly_119_) and the heavy (_207_Asp–Arg_370_) and the light (_370_Asp–Leu_439_) chains of the papain-like catalytic domain [4] (Scheme 1). These three tightly associated domains are held together by non-covalent interactions. The N-terminal exclusion domain is a unique structural feature among the papain family responsible for the diaminopeptidase activity. This occurs through the blockage of the active site beyond the S2 pocket [5]. Processed dimers finally associates non-covalently to form a physiologically active homo-tetramer of ~200 kDa [5].

Genetic forms of CatC deficiency are associated with Papillon–Lefèvre syndrome (PLS, *OMIM* No. 245000) or Haim–Munk syndrome (HMS, *OMIM*. No. 245010) in humans [6,7,8]. PLS and HMS are characterized by palmoplantar keratoderma and severe periodontitis. However, a number of additional findings have been reported in HMS, including arachnodactyly, acro-osteolysis, atrophic changes of the nails, and a radiographic deformity of the fingers [8]. There is a general consensus that CatC is primarily responsible for the maturation of granule-associated hematopoietic serine proteinase zymogens, including neutrophil elastase, proteinase 3, cathepsin G [9], and NSP4 [10], with pro-inflammatory and immune functions [11]. The role of CatC in zymogen activation has been clearly shown in humans and mice with CatC deficiency, which results in severe reduction or abolition of proteolytic activity of its target proteinases [9,12,13]. Hence, CatC has been proposed as a valuable druggable target for the treatment of neutrophilic proteinase-driven pathologies such as chronic obstructive pulmonary disease (COPD), cystic fibrosis, granulomatosis with polyangiitis, and rheumatoid arthritis [11,14,15,16].

CatL, and to a lesser extent CatS, have been identified as proCatC maturating proteinases in vitro [4], but are not required for CatC activity in mice [17]. We have recently studied the maturation of proCatC in human neutrophilic precursor cells [18]. We did not succeed, however, in totally blocking the maturation of CatC using a cell-permeable pharmacological inhibitor of CatS [18]. In this work, we investigated the maturation of human recombinant proCatC by related cysteine cathepsins that have a very similar proteolytic activity and substrate specificity to CatL and CatS. In parallel, a structural analysis of proCatC by molecular modeling was performed to strengthen our experimental results. Finally, we investigated the stabilizing role of the CatC propeptide using a recombinant proCatC that displayed the single Leu172Pro mutation (Scheme 1), mimicking a missense mutation identified in a PLS patient [19] and in an HMS patient [20]. 

## 2. Results

### 2.1. Processing and Maturation of Wt-proCatC by CatK, CatV, or CatF

**Figure 1:**
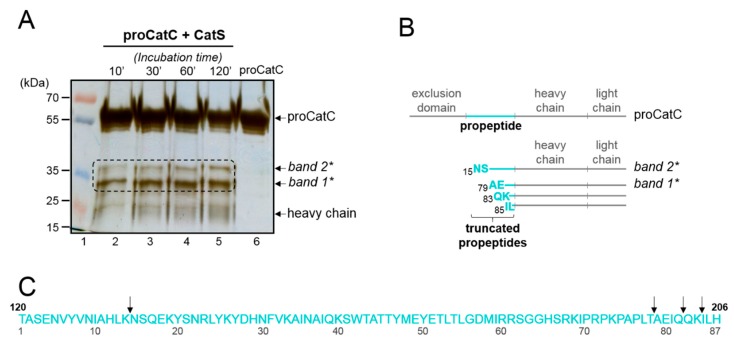
Wt-proCatC maturation by CatS. (**A**) ProCatC (50 µM) processing by CatS (1 µM) in vitro. The progress of the processing was analyzed after 10 min (lane 2), 30 min (lane 3), 60 min (lane 4), and 120 min (lane 5) by SDS-PAGE and silver staining. Molecular mass standards and control of proCatC are shown in lanes 1 and 6, respectively. (**B**) Structure of proCatC and cleavage products carrying truncated propeptides at the N-terminus after the initial cleavages of proCatC by CatS. The N-terminal residues of band 2* and band 1* were identified by Edman sequencing. Band 2* and band 1* contain heavy and light chains, as shown in Reference [18]. (**C**) Amino acid sequence of the CatC propeptide (residues 120–206) in single letter code. Arrows indicate the processing sites of CatS, as determined by Edman sequencing of band 2* and band 1.

**Figure 2:**
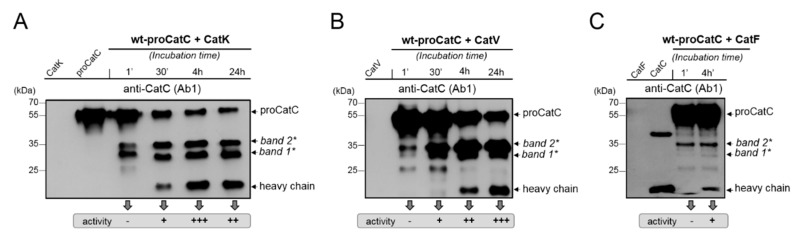
Wt-proCatC maturation by CatK, CatV, and CatF. Western blot analysis of endoproteolytic cleavage of proCatC by recombinant cathepsins using an anti-CatC Ab. Wt-proCatC (2 μM) was incubated at 37 °C for different time intervals with (**A**) human CatK (0.1 µM), (**B**) human CatV (0.1 µM), and (**C**) human CatF (0.5 µM). The progress of the cleavage reaction was monitored by western blot detection and by measurement of CatC activity. The activity box gives an approximation of the CatC activity as measured using a CatC-selective fluorogenic substrate. Similar results were obtained in three independent experiments.

The recombinant precursor of CatC, proCatC is processed and converted to its mature form by CatL [4,14] and CatS [18] in two consecutive steps (Step 1, processing of the propeptide; Step 2, processing of the catalytic papain-like domain) in vitro. N-terminal amino acid sequencing of the 36 kDa peptide (band 2*) generated by CatS (Figure 1A) after transfer on PVDF membrane revealed a unique CatS cleavage site (--LK_14_^↓^N_15_SQE--) within the proregion (Figure 1A), whereas several cleavages were identified for the N-terminus of the 33 kDa peptide (band 1*) within the Thr78 to Ile85 segment close to the C-terminal end of the propeptide (Figure 1B,C). The ability of both human CatV and CatK, which share related substrate specificity with CatL and CatS, to process proCatC was first assayed in vitro. Indeed, incubation of wt-proCatC with CatV or CatK yielded similar processed forms. Moreover, concomitant hydrolysis of Gly–Phe–AMC revealed that proCatC was indeed converted into its catalytically active mature form (Figure 2A,B). This processing was initiated by the release of two intermediate peptides (i.e., 36 kDa band 2* and 33 kDa band 1*) (Figure 2A,B) resulting from cleavages at the N- and C-termini of the propeptide (Step 1). Supplementary processing between the heavy and light chains led to the mature and proteolytically active CatC (Step 2) (Figure 2A,B). Accordingly, these in vitro data support our suggestion that the Cat L/S related cathepsins CatV and CatK may also process and activate proCatC during biosynthesis. In addition, the rates of hydrolysis after conversion by CatV and CatK were comparable to those by CatL and CatS. By contrast, activation of proCatC by CatF was slower and less efficient (Figure 2C). Calpain-1, a Ca^2+^-dependent cytosolic cysteine endopeptidase, trypsin, and CatG hydrolyzed proCatC, but failed to generate a mature and proteolytically active CatC (Supplementary Figure S1). 

### 2.2. Structural Modeling of Wt-proCatC

**Figure 3:**
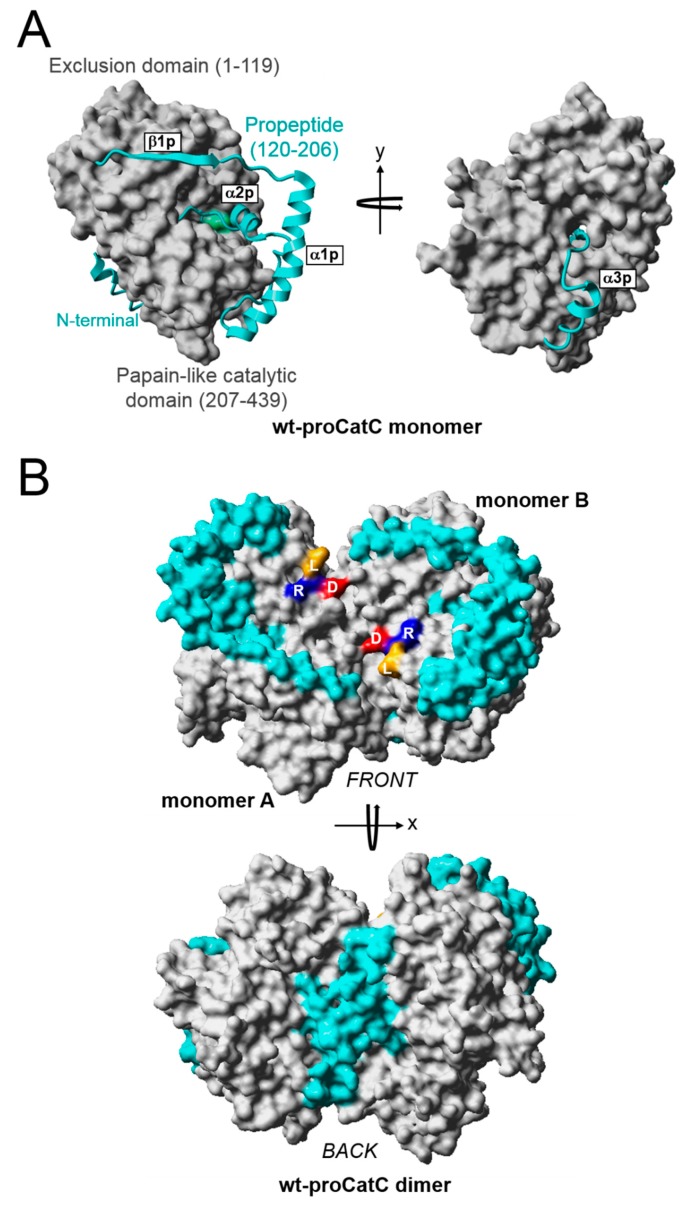
Model structure of wt-proCatC. (**A**) ProCatC monomer. The exclusion domain and papain-like catalytic domain are shown in surface representation and colored in grey. The propeptide (residues Thr120–His206) in the ribbon plot is shown in cyan. The catalytic cysteine 234 is shown in green. (**B**) ProCatC dimer. Monomers A and B are shown in surface representation with the same color coding as in (**A**). Leucine, arginine, and aspartic acid are labeled using their one-letter amino acid codes and shown in dark yellow, blue, and red, respectively.

The crystal structures of human mature CatC and that of proCatB from *Trypanosoma brucei* share similar structural features with human proCatC, and were therefore used to model and position the two propeptides within a proCatC dimer and to identify proteinase-sensitive sites [21]. The proCatC propeptide (residues Thr120–His206) in each monomer of the stable dimeric proCatC consisted of a N-terminal β-strand (β1p = 1p–8p) and three α-helices (α1p = 14p–39p, α2p = 54p–62p, α3p = 77p–87p), helices α1p and α2p being connected by 15 residues (residues Lys159–Thr173) (Figure 3A,B). The propeptide binding loop (PBL)–propeptide interface formed a stable two-stranded β-sheet. Hydrophobic side chain contacts and hydrogens bonds stabilized the PBL–propeptide interface. The C-terminal portion of the pro-segment contained the helix α3p. We observed symmetrical contacts (less than 4.5Å) between the two stabilized chains of the dimeric model structure, most of them being between the exclusion domain of one monomer and the light chain of the other (Table 1). Propeptide Leu196 residues were close to one another and Thr197, located in the vicinity of His59 from the exclusion domain. The residues Gln201, Ile204, Leu205, and Pro208 from the C-terminal part of the propeptide localized in the monomer interface might also participate in the dimerization process (Table 1).

**Figure 4:**
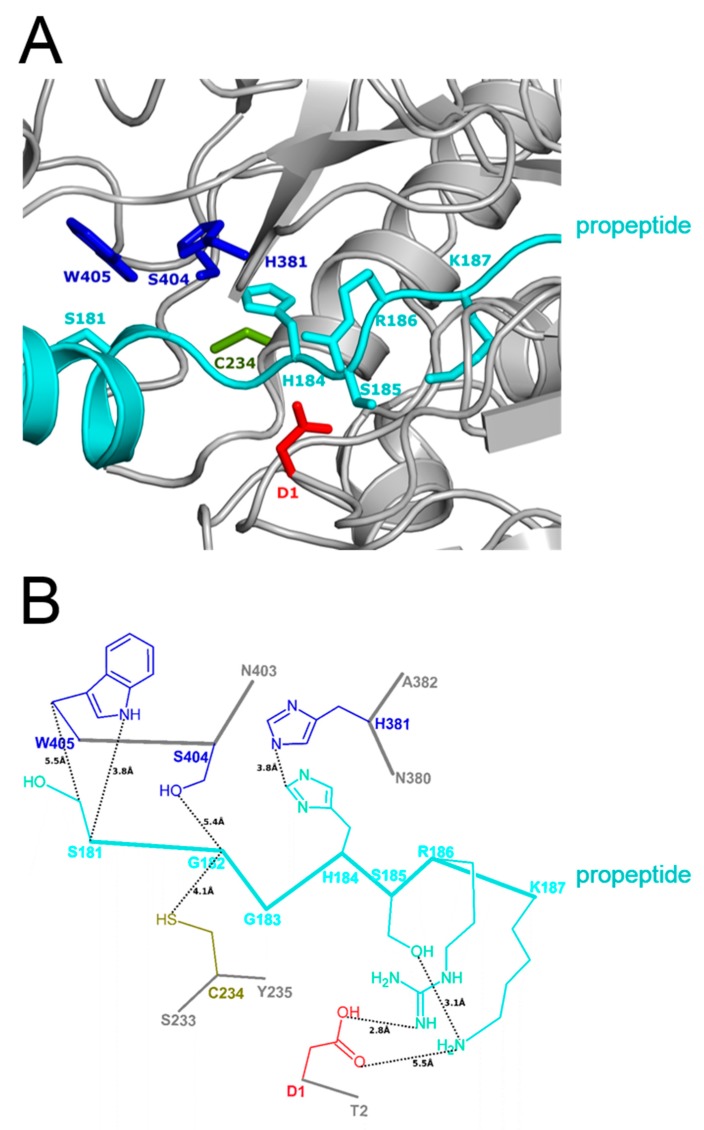
Interactions of propeptide with CatC within the substrate-binding cleft. (**A**) Ribbon presentation of the enzyme (gray) contact with the propeptide (cyan). Residues 181–187 of the propeptide are shown in full. Exclusion domain, propeptide, heavy chain, and light chain residues are shown in red, cyan, green, and blue, respectively. (**B**) The hydrogen bonds between the propeptide and the enzyme residues are shown in a dashed black line with the same color coding as in (**A**). Residues are labeled using their one-letter amino acid code.

A concave surface was formed in the proCatC dimer lined by the solvent-exposed propeptide segment (residues 120–162) of each monomer. The sequences containing the cleavage sites of proCatC maturating proteinases (_369_Leu–Arg^↓^Asp_371_) between the heavy and light chains were located at the bottom of this concave structure and seemed to be inaccessible to proteinases (Figure 3B). Propeptide residues 181–187 were located in the active center of CatC, with Gly182 in the center of the active site (Figure 4A). Only small residues like glycine or alanine could fit in this area because of the narrow space. The carbon α of Gly183 was directed towards the empty space of the protein. His184 interacted with His381 to form the active site. We observed also a salt bridge between Arg186/Lys187 from the propeptide and Asp1 of CatC (Figure 4B).

During maturation, the structure of proCatC in the dimer underwent several modifications resulting in the removal of the propeptide (band 1*, Step 1), which was not the case in the rigid model presented in Figure 1; Figure 2. Further simulations allowing more flexibility of the propeptide within the proCatC dimer showed that interactions between each propeptide and the activation domain and those between the two propeptides could be abolished while the dimeric structure of proCatC was preserved. Thus, the dimer would oscillate between two conformations, one of which opened access to processing proteinases that could remove the propeptide from the inactive dimer (Supplementary Figure S2, videos 1,2). Further, the equilibrium between a “closed” and an “open” conformation did not compromise the association of the exclusion domain to the core of the zymogen. The salt bridge between Arg186/Lys187 and Asp1 remained during conformational changes.

### 2.3. Characterization and Structural Modeling of ProCatC(Leu172Pro) 

**Figure 5:**
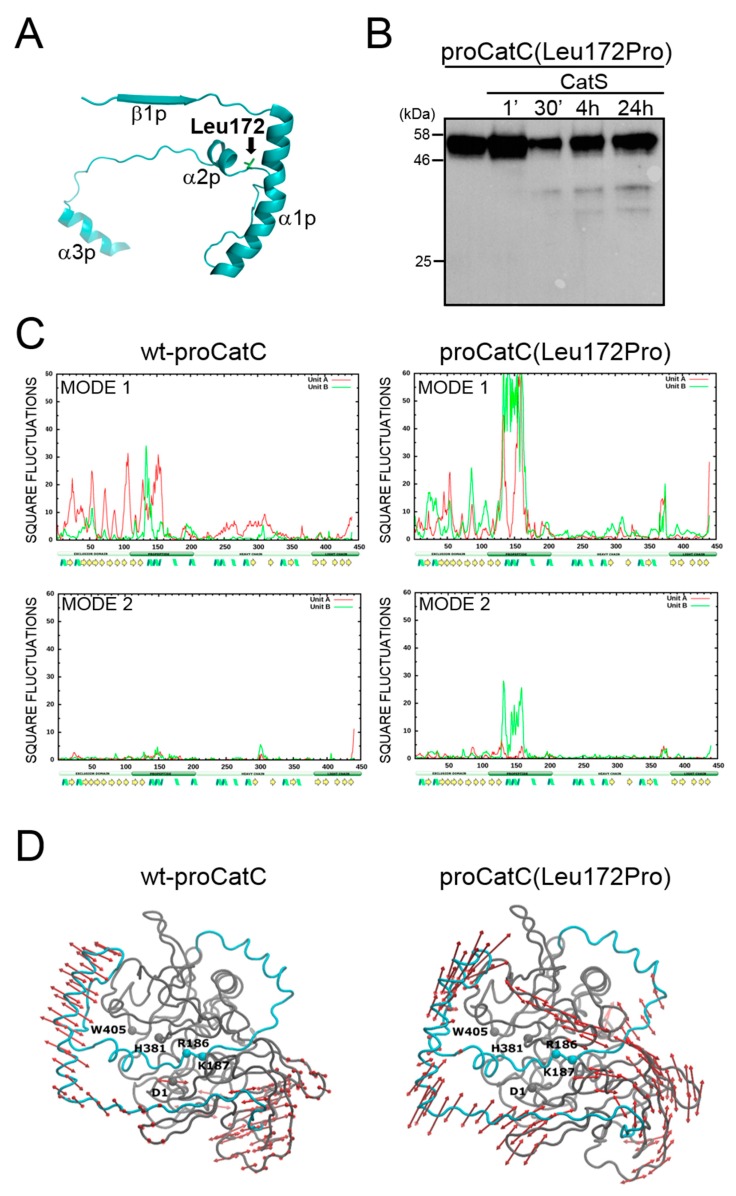
Production, processing and principal component analysis (PCA) of proCatC(Leu172Pro). (**A**) Model structure of proCatC propeptide in ribbon plot, showing the location of Leu172. (**B**) Western blot analysis of proCatC(Leu172Pro) produced in HEK293 cells and incubated with CatS. Purified recombinant proCatC(Leu172Pro) was incubated at 37 °C for different time intervals with human recombinant CatS. Similar results were obtained in three independent experiments. (**C**) PCA analysis of wt-proCatC and ProCatC(Leu172Pro). (**D**) Model structures of wt-proCatC and proCatC(Leu172Pro) monomers. Red: exclusion domain, cyan: propeptide, green: heavy chain, blue: light chain. The arrows are the eigenvectors as calculated by using the first eigenvalue of the covariance matrix.

The Leu172Pro mutation in the CatC zymogen propeptide (Figure 5A), which is associated with an impairment of proCatC activation into proteolytically active CatC, has been found in patients suffering from PLS and HMS [19,20]. Here, we took advantage of this characteristic clinical observation to tentatively delineate how this non-conservative missense mutation in the propeptide affects the maturation of proCatC. First, we cloned and expressed proCatC(Leu172Pro) in human embryonic kidney 293 cells (HEK293 EBNA). ProCatC(Leu172Pro) was produced as a soluble secreted protein, but with a lower yield compared to wt-proCat, consistent with our finding that the mutant was preferentially located as an insoluble protein in inclusion bodies inside the cells (data not shown). By contrast to wt-proCatC, purified proCatC(Leu172Pro) was mostly found in a monomeric form rather than as a dimer, according to gel permeation chromatography. Following incubation with CatS, faint amounts of two peptides with Mr(app) of ~36 and ~33 kDa were generated from proCatC(Leu172Pro) (Figure 5B), as observed with wt-proCatC. The proteolytic release of the heavy and the light chains from each peptide, i.e., the second maturation step, however, was impaired indicating a defective processing. The modeled structure of the mutant revealed that the Leu172Pro mutation had a strong influence on the folding of the propeptide in comparison to wt-proCatC, especially on the segment between residues 160–170. In case of the wt sequence, we only saw a minor fluctuation of the affected segment; however, in case of the Leu172Pro mutant, the fluctuations were much larger and could be seen on the plots (Figure 5C) and on the modeled structure (Figure 5D).

## 3. Discussion

As with other lysosomal cysteine cathepsins, CatC is expressed as an inactive zymogen. The sequence of proCatC, however, is partitioned into three instead of two domains, an exclusion domain, a propeptide, and a C-terminal catalytic domain with a papain-like structure. Another unique feature of proCatC is the spontaneous formation of dimers [4]. The N-terminal part of proCatC, which contains both the exclusion domain and the propeptide, has been proposed to act as an intramolecular chaperone that assists in proper zymogen folding [4]. Maturation of the proCatC dimer is achieved by the proteolytic removal of its internal propeptide segment and cleavage of the catalytic domain into a heavy and light chain. The 3D zymogen structures of other cysteine cathepsins show that their propeptide segments fold on the surface of the catalytic domain in an extended conformation running through the active site cleft in the opposite direction to the substrate, blocking access to the active site [2,22,23]. Using homology modeling, we obtained a similar fold for the proCatC propeptide as that found in other procathepsins. The propeptide occludes the active site region and is stabilized by a salt bridge between Arg186/Lys187 and the carboxylic group of the conserved Asp1 side chain, which is responsible for the anchoring of the N-terminal amino group of CatC substrates [5]. Symmetrical contacts, which are stabilized by interactions between the exclusion domain of one monomer and the light chain of the other one within the dimer, were identified between the two C-terminal propeptide segments. The C-terminal propeptide segments involved in the dimerization are located on the surface of zymogen dimers, thus preventing their assembly into zymogen tetramers, as previously suggested [4].

The maturation of human proCatC by both human CatL [4,14] and CatS [18] implies two consecutive steps. We previously showed that CatS first hydrolyzes peptide bonds within the propeptide sequence either in the N-terminal region (--HL**K**_14_^↓^NSQ--), generating a peptide of Mr(app) ≈36kDa (band 2*), or in the C-terminal region (after Thr78, Gln82, and Lys84; --PL**T**_78_^↓^AEI**Q**_82_^↓^Q**K**_84_^↓^ILH, respectively) generating a peptide of Mr(app) ≈33 kDa (band 1*) [18]. In agreement with these cleavage positions, Turk and coworkers [4] reported that cleavages by CatL occurred at the N-terminal (--HL**K**_14_^↓^NSQ--) and C-terminal ends of the propeptide (--PL**T**_78_^↓^AEIQQ**K**_84_^↓^ILH). These hydrolysis-sensitive sequences are located in close vicinity to the boundaries of two α-helices (residues 24–64), consistently with previously reported cell-based experiments, suggesting that these hinge sequences are highly susceptible to proteolysis [18]. The second step occurs in the catalytic domain and generates the final heavy and light chains [4,14,18]. Only after this processing of the catalytic domain is CatC activity generated, indicating that the cleavage between heavy and light chains is required for the final active conformation.

CatV and CatK are closely related to CatL and CatS, respectively. Both display a highly conserved active site region, a relatively broad CatL-like substrate specificity, and overlapping endopeptidase activities [1,2,3]. We have identified CatV and CatK as putative proCatC maturating proteinases, since they generate intermediate peptides of Mr(app) similar to those obtained with CatL and CatS and release active CatC with an efficiency similar to that of CatL or CatS. CatF shares about 42–43% homology with CatK, CatL, and CatS, and possesses a crystal structure similar to that of CatL-like proteinases [24]. Although it displays a similar specificity and activity on small synthetic substrates as CatL-like proteinases, it activates proCatC less efficiently than cathepsins L, S, V, and K [24,25]. The reason why diverse cathepsins can maturate proCatC has not yet been elucidated, but probably reflects their partially redundant preferences [26]. One might also argue that the distribution of CatC and activating proteinases differs between cells and tissues.

Our model structure showed that a concave surface in the vicinity of the active sites was formed in the proCatC dimer, lined by the solvent-exposed propeptide segments of each monomer. The sequences containing the cleavage sites of CatL or CatS were located at the bottom of this concave structure and appeared to be non-accessible. The presence of the propeptide segments could explain the sequential maturation of proCatC in two steps. Processing and removal of the propeptide could, however, hardly occur if the dimer was stabilized by propeptide–propeptide and propeptide–exclusion domain interactions. In view of these considerations, one must assume that the proCatC dimers stand in a dynamic equilibrium with an “open” and a “closed” conformation, the former initiating the processing and the removal of the propeptide. Rebernik and colleagues reported recently that soluble recombinant human proCatC monomers without the exclusion domain were incapable of forming dimers [27], supporting the involvement of the exclusion domain in dimer formation. After removal of the propeptides in the first step, CatC dimers could form a non-stable tetramer in a mixture of dimeric and tetrameric states. The processing between heavy and light chains of the catalytic domain catalyzed specifically by CatL, CatS CatK, CatV, and CatF in the second step is essential for achievement of active CatC. All these data suggest that the tetrameric structure of CatC built after several rounds of processing helps to stabilize the exclusion domain in the right position to confer upon CatC its aminopeptidase activity.

Several loss-of-function missense mutations in the CatC gene *CTSC* have been identified in patients with PLS or HMS [28]. The structure-based interpretations of some missense mutations helped to understand the loss of CatC activity by disturbing the oxyanion hole, the binding of the chloride ion, or the disulfide connectivity [5]. The Leu172Pro mutation identified in patients suffering from PLS or HMS was located on the propeptide of CatC [19,20]. Thus, it was conceivable that the Leu172Pro mutation prevented proper folding of the propeptide, disturbing dimer formation. We observed that the mutation altered the processing of proCatC(Leu172Pro) by CatS and blocked its maturation. The Leu172Pro mutation caused a conformation alteration of the propeptide fragment 160–170, as a proline residue can disrupt the organization of the backbone of polypeptides. The conformation of the propeptide fragment 160–170 probably plays some important role in the conformation and the maturation of proCatC. Sorensen et al. observed that the missense mutation Tyr168Cys in the propeptide region of CatC resulted in complete disappearance of the mutant in purified neutrophils collected from a PLS patient [29]. Pulse-chase biosynthesis performed with immature bone marrow myeloid cells from the PLS patient demonstrated that CatC was correctly expressed [29]. The absence of the mutant CatC in mature PLS patient bone marrow cells suggested that the mutant was degraded prior to this stage. It is conceivable that the introduction of a Cys in the propeptide region of CatC could result in the formation of an aberrant inter- or intra-protein disulfide bond, which could alter the conformation and the stability of the zymogen. Intracellular degradation of CatC carrying a missense mutation has been suggested to explain the loss of CatC in blood cell lysates or in the urine of PLS patients [12,30]. The likely degradation of proCatC carrying a missense mutation in the propeptide region is clearly supported by the fact that the amount of recombinant proCatC(Leu172Pro) produced in HEK293 cells was much lower than that of wt-proCatC. 

We conclude that the propeptide of proCatC is involved in the formation of a stable dimer and in the sequential maturation process of proCatC, which can be endoproteolytically catalyzed by several cathepsins. We identified CatF, CatK, and CatV as new proCatC activating candidates in addition to CatS and CatL. ProCatC processing and maturation is apparently quite variable in terms of biosynthetically relevant proteinases. The identification of proteinases capable of converting proCatC to its active tetrameric form, and the subcellular localization of these candidate proteinases in comparison to CatC in various tissues, will contribute to a detailed understanding of proCatC activation in vivo. 

## 4. Materials and Methods

### 4.1. Enzymes and Reagents

Human CatC was from Unizyme (Hørsholm, Denmark). Human CatS and CatK were supplied by Calbiochem (VWR International, Pessac, France). Human recombinant CatV and proCatF came from BPS Bioscience (San Diego, USA). The murine anti-human CatC antibody (Ab1, antiCatC D-6, sc-74590) and the peroxidase-conjugated anti-mouse IgG (#A5906) were from Santa Cruz Biotechnology (Heidelberg, Germany) and Sigma-Aldrich (Saint-Quentin-Fallavier, France), respectively. Fluorogenic substrates Z-Phe-Arg-AMC (sc-3136) and Gly-Phe-AMC were purchased from Santa Cruz biotechnology (Heidelberg, Germany) and MP Biomedicals (IllKirch, France), respectively.

### 4.2. Structural Modeling of Human Wt-proCatC and ProCatC(Leu172Pro)

The model was based on the crystal structure of human CatC (Protein Data Bank (PDB) code 3PDF, [31]) and the crystal structure of trypanosoma brucei proCatB (PDB code 4HWY, [21]). The dimer was constructed by the application of the symmetry operator present in the PDB file. The middle fragment of propeptide was modeled using SWISS-MODEL server (https://swissmodel.expasy.org/). All missing loops, connecting the propeptide with the other chains, were then added using Yasara program (www.yasara.org). The obtained model was refined by coarse-grained replica exchange molecular dynamics simulation with a UNRES force field [32]. During the simulation, secondary structure and positional (applied for Cα atoms) restraints (based on two 3PDF and 4HWY), were applied. Finally, to obtain a low energy, all-atom structure, the model was optimized using minimization and short, low temperature molecular dynamics with Generalized Born implicit solvent in the AMBER package (http://ambermd.org/). This procedure was repeated in cycles until all close contacts were optimized. To keep the model structure as close as possible to the experimental structures, the positional restraints for carbon α atoms of 2 kcal/mol.Å^2^ were used during all-atom simulations. All models were analyzed using RasMol AB software [33]. All molecular dynamics simulations were performed for neutral pH using AMBER and UNRES force fields, since the force field parametrization was made to imitate the behavior of amino acids at pH around 7.

### 4.3. Principal Component Analysis (PCA) of Human Wt-proCatC and ProCatC(Leu172Pro)

To observe differences in the molecular motions, wt-proCatC and proCatC(Leu172Pro) mutant were simulated independently. In both cases, the dimer (each of unit contained 441 residues) was computed. To neutralize negative charge on the molecule, 8 Na^+^ ions were added. The model was immersed in a TIP3P water box (about 38.000 water molecules) of approximately 115 × 85 × 125 Å. The newly built molecular systems were simulated for 100 ns using molecular dynamics at the temperature of 310 K, to distinguish the differences in molecular motions of wt-proCatC and proCatC(Leu172Pro). To distinguish the differences in molecular motions of wt-proCatC and proCatC(Leu172Pro), principal component analysis (PCA) was used. PCA is the method which allows for the decomposition of the molecular motions as observed in the molecular dynamics simulation [34]. The two modes with the highest influences are shown in Figure 5. Mode 1 described about 55–65% of all protein motions. Mode 2 described about 5–10%. Mode 1 described the mutual orientation of the protein domains (opening). Mode 2 described the local rearrangement of the exclusion domain.

### 4.4. Cloning, Production, and Purification of Recombinant ProCatC Proteins

Production of recombinant mature human proCatC with a C-terminal hexa-histidine tag was carried out by transfection of human embryonic kidney (HEK293 EBNA) cells (Scheme 1). Human proCatC cDNA was ordered from Integrated DNA Technologies (Coralville, IO, USA). The cDNA was then cut with KpnI and AgeI (New England Biolabs, Ipswich, MA, USA) and ligated into the pTT5 vector (NRC Biotechnology Research Institute, Ottawa, ON, Canada) linearized with the same enzymes. After transformation of competent *Escherichia coli* DH5α, positive clones were purified with an EndoFree Plasmid Maxi Kit (Qiagen, Hilden, Germany) and screened by DNA sequencing in Eurofins MWG GmbH (Ebersberg, Germany). Prior to transfection of HEK293 EBNA with the expression constructs, cells were brought to a density of 1 × 10^6^ cells/mL. Polyethyleneimine (PEI, Invitrogen, Carlsbad, CA, USA)/DNA complexes were prepared by adding PEI to DNA, both prediluted in Optipro serum-free medium (Invitrogen, Carlsbad, CA, USA), incubated at room temperature for 20 min, and added to the cells. The transfection of HEK293 EBNA cells was performed with 2 μg of PEI and 1 μg of DNA for 1 mL of cell culture suspension. After 1 day, Bacto TC Lactalbumin Hydrolysate (BD Biosciences, Franklin Lakes, NJ, USA), was added as an amino acid supplement to 0.5% final concentration. Supernatants were harvested after 4 days.

The proCatC(Leu172Pro) was produced with an extra C-terminal hexa-*histidine* tag to differentiate it from the wild-type counterpart. The site-specific mutation Leu172Pro was introduced to the wt-proCatC by cDNA exchange. Integrated DNA Technologies (Coralville, IO, USA) provided us the cDNA (sequence: GAGGGTTCCAAGGTAACCACATATTGCAATGAAACAATGACTGGTTGGGTGCATGATGTCCTAGGCCGGAACTGGGCATGCTTCACGGGGAAAAAAGTTGGTACAGCGAGCGAGAACGTTTATGTTAACATCGCACACTTGAAAAATAGTCAGGAGAAGTACTCCAATAGGCTGTACAAGTACGACCATAACTTTGTAAAAGCTATCAATGCGATACAAAAATCCTGGACAGCTACTACATACATGGAGTACGAGACTCTTACTTTGGGGGATATGATAAGAAGGTCTGGTGGACACAGTAGGAAGATTCCGAGACCTAAACCCGCACCCCCTACGGCTGAAATCCAACAAAAAATACTCCATCTCCCCACGTCCTGGGATTGGCGCAATGTCCACGGCATAAACTTTGTGTCCCCCGTTCGAAATCAAGCT). The cDNA was further treated by BstBI and AvrII (New England Biolabs, Ipswich, USA) and ligated into the previous pTT5 vector with wt-proCatC, already linearized with the same enzymes. After transformation of competent *E. coli* DH5α, positive clones were purified and screened as the wt-proCatC. The transfection of HEK293 EBNA cells was performed with 2 μg of PEI and 0.1 μg DNA per mL of cell culture suspension, as previously described for the wild-type enzyme.

After 4 days, the cell culture was centrifuged for 5 min RT at 300 rpm. Finally, the supernatant of proCatC proteins was collected and filtered (0.22 µm), and then dialyzed against 20 mM disodium phosphate, 300 nM sodium chloride, and 5 mM imidazole, pH 7.4 and loaded onto a HisTrap column (GE Healthcare Life Sciences, Buckinghamshire, UK) equilibrated with 20 mM Tris-HCl, 500 nM sodium chloride, and 5 mM imidazole, pH 7.4 (AKTA chromatographic system, GE Healthcare Life Sciences, Buckinghamshire, UK) (flow rate: 1 mL/min). The column was washed exhaustively before removal of bound proCatC proteins by a linear imidazole gradient (5 mM–1 M). Proteins were assayed with a bicinchoninic acid assay (BCA) (Thermo Ficher Scientific, Villebon sur Yvette, France).

The purity of each proCatC preparation was assessed by Coomassie staining and western blotting under denaturing/reducing conditions. The samples were separated by SDS-PAGE and transferred to Hybond-ECL membranes. Free sites were saturated by incubation in PBS, 0.1% Tween, and 5% fat-free milk for 1 h, and the membrane were then incubated with anti-CatC antibody (diluted 1:800) in PBS, 0.1% Tween, and 5% nonfat milk overnight at 4 °C. The membranes were washed three times in PBS, 0.1% Tween and incubated for 1 h with peroxidase-conjugated anti-mouse IgG (diluted 1:10.000) (Sigma-Aldrich), a peroxidase-coupled secondary antibody diluted in PBS, 0.1% Tween, and 5% nonfat milk. Reactive bands were identified by chemiluminescence (ECL Plus Western Blotting Kit Detection Reagents, GE Healthcare, UK) according to the manufacturer’s instruction.

### 4.5. Processing of Human ProCatC Proteins

Purified proCatC (50 μM) was incubated for 0–120 min in the presence of E64 titrated CatS (0.1–1 μM). The reaction was carried out at 37 °C in 50 mM sodium acetate buffer pH 5.5, containing 30 mM NaCl, 1 M EDTA, and 1 mM DTT. The same experiment was performed for proCatC in the presence of titrated CatK (0.1 μM) in 20 mM citric acid, 150 mM NaCl, 10 mM DTT, and 1 mM EDTA, pH 4.5; CatV (0.1 μM) in 25 mM sodium acetate buffer pH 5.5, 100 mM NaCl, and 5 mM DTT; and CatF (0.5 μM) in 100 mM sodium acetate buffer pH 5.5, containing 2 mM EDTA, 8 mM DTT, and 0.05% Brij35. ProCatF was processed for 90 min as mature active CatF in 50 mM sodium acetate buffer pH 4.5 in the presence of 5 mM DTT at RT prior use, according to the supplier recommendations (BPS Bioscience). CatF activity was quantified with the fluorogenic substrate Z–Phe–Arg–AMC.

ProCatC maturation was monitored by western blot, as detailed in the previous paragraph. Alternatively the proteolytic activity of mature CatC was monitored at 37 °C in 50 mM sodium acetate buffer pH 5.5, containing 30 mM NaCl, 1 M EDTA, and 1 mM DTT, using Gly–Phe–AMC as a fluorogenic substrate (spectromicrofluorimeter SpectraMax Gemini, Molecular Devices, Saint Grégoire, France; λ_ex_ = 350 nm, λ_em_ = 460 nm). Identification of the cleavage sites was done by Edman sequencing (automated protein sequenator, Procise P494-Applied Biosystems). The same experiments were repeated for the punctual mutant of wt-CatC, proCatC(Leu172Pro) (50 μM).

## Figures and Tables

**Table 1:** Residues localized in monomer interaction interfaces.

Chain A/B	Chain B/A
Y8	D371, N374
L9	D371
Q45	N332, E333
K46	G325 (backbone)
L47	L47, F327, N374
T49	L335
Y51	E333, A334
N56	L205
S57	Q201
H59	T197
L196	L196
F327	F327

Exclusion domain, propeptide, heavy chain, and light chain residues are shown in red, cyan, green, and blue, respectively. Weak interactions were observed with Pro208 and G55. I204 could weakly interact with S57 and G55.

## Supplementary Materials

Supplementary materials can be found at https://www.mdpi.com/1422-0067/20/19/4747/s1. Supplementary Figure S1: ProCatC dimers in an “open” and a “closed” conformation. Supplementary Figure S2: ProCatC processing by calpain-1, trypsin and CatG. Videos: ProCatC dimers in a dynamic equilibrium with an “open” and a “closed” conformation. The activation domain is colored in red. PDB files of proCatC model structures (monomer and dimer).

## Abbreviations

Cat	Cathepsin
HMS	Haim–Munk syndrome
HEK	HEK, human embryonic kidney
PLS	PLS, Papillon–Lefèvre syndrome
PDB	PDB, Protein Data Bank
Enzymes	Cathepsin C (EC 3.4.14.1), cathepsin F (EC 3.4.22.41), cathepsin K (EC 3.4.22.38), cathepsin L (EC 3.4.22.15), cathepsin S (EC 3.4.22.27), cathepsin V (EC 3.4.22.43)
