# Supplementary material for: Processing and Maturation of Cathepsin C Zymogen: A Biochemical and Molecular Modeling Analysis
Source: Int J Mol Sci. 2019 Sep 25;20(19):4747. doi: 10.3390/ijms20194747 (PMC6801622; doi:10.3390/ijms20194747)

**SUPPORTING INFORMATION**

- **Supp.Figure 1**: ProCatC dimers in an “*open”* and a “*closed”* conformation.
- **Supp.Figure 2**: ProCatC processing by calpain-1, trypsin and CatG
- **Videos**: ProCatC dimers in a dynamic equilibrium with an “*open”* and a “*closed”* conformation. The activation domain is colored in red.
- **PDB files**.

**SuppFIGURE 1: Wt-proCatC dimers in a “*closed”* and an “*open”* conformation.**


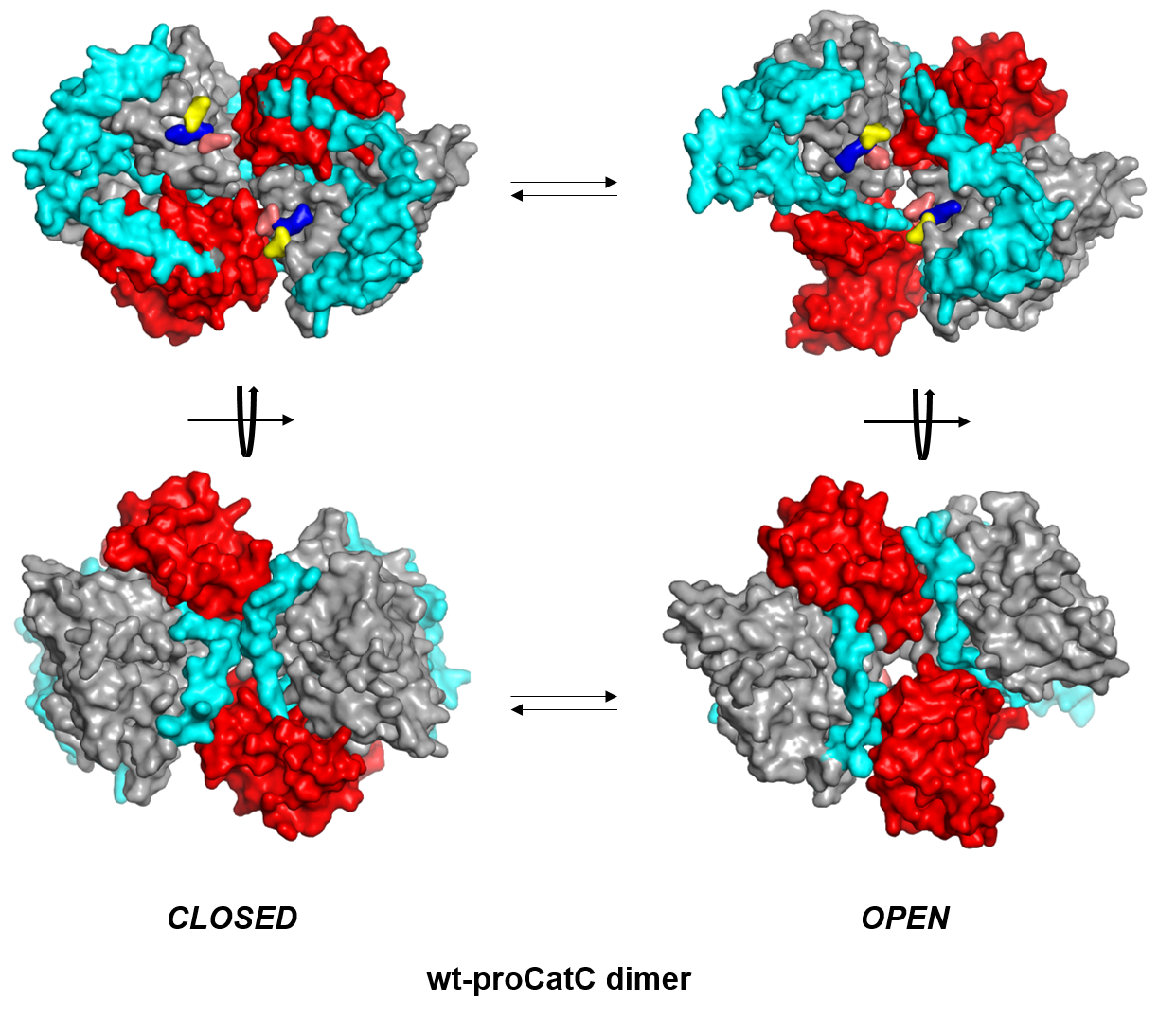


**SuppFIGURE 2: Wt-proCatC processing by calpain-1, trypsin and CatG.** Western blot analysis of wt-proCatC processing by cysteine and serine endoproteases using an anti-CatC Ab. Wt-proCatC was incubated with **A**) human calpain-1 (0.5 μM) in 50 mM Hepes buffer pH7.5, containing 1 mM NaN3, 5 mM β-mercaptoethanol, 2 mM CaCl_2_, **B**) bovine trypsin (0.5 µM) in PBS, **C**) with human CatG (0.5 µM) in 50 mM HEPES buffer pH7.4, containing 750 mM NaCl, 0.05% Nonidet P-40, at 37°C for increasing time periods. CatC processing was analyzed by Western blot and by measurement of CatC activity. Similar results were obtained in three independent experiments using an anti-CatC Ab. A major peptide of 33 kDa was noticed when proCatC was incubated with calpain-1. When proCatC was incubated with trypsin or CatG, peptides of ~36 and ~33 kDa were generated. The appearance of the peptides of ~36 kDa and ~33 kDa confirmed the presence of highly proteolysis-sensitive segments at the N and C-terminus of the CatC propeptide.


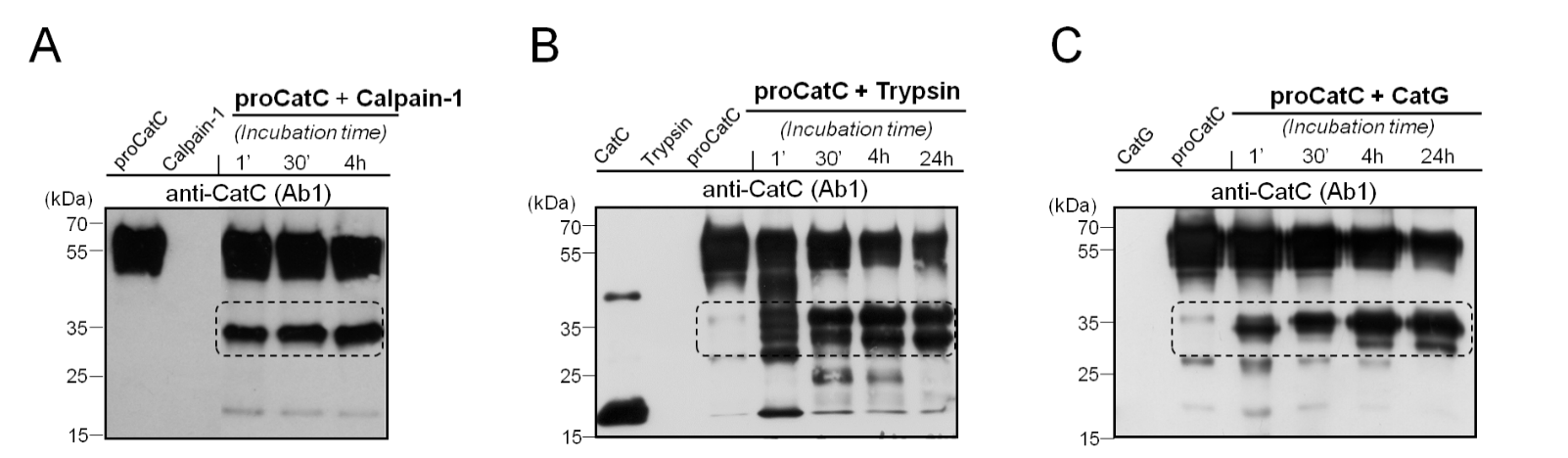

Supplement: Supplementary file 1 [file ijms-20-04747-s001.zip › ijms-587176-supp/ijms-587176-supp.docx]
